# High viral loads: what drives fatal cases of COVID-19 in vaccinees? – an autopsy study

**DOI:** 10.1038/s41379-022-01069-9

**Published:** 2022-04-01

**Authors:** Klaus Hirschbühl, Tina Schaller, Bruno Märkl, Rainer Claus, Eva Sipos, Lukas Rentschler, Andrea Maccagno, Bianca Grosser, Elisabeth Kling, Michael Neidig, Thomas Kröncke, Oliver Spring, Georg Braun, Hans Bösmüller, Maximilian Seidl, Irene Esposito, Jessica Pablik, Julia Hilsenbeck, Peter Boor, Martin Beer, Sebastian Dintner, Claudia Wylezich

**Affiliations:** 1 Hematology and Oncology, Medical Faculty, University of Augsburg, Augsburg, Germany; 2 General Pathology and Molecular Diagnostics, Medical Faculty, University of Augsburg, Augsburg, Germany; 3 Microbiology, Medical Faculty, University of Augsburg, Augsburg, Germany; 4 Medical Clinic, University Hospital Augsburg, Augsburg, Germany; 5 Diagnostic and Interventional Radiology, Medical Faculty, University of Augsburg, Augsburg, Germany; 6 Anesthesiology and Operative Intensive Care Medicine, Medical Faculty, University of Augsburg, Augsburg, Germany; 7 Gastroenterology, Medical Faculty, University of Augsburg, Augsburg, Germany; 8 Institute for Pathology and Neuropathology, University Hospital of Tübingen, Tübingen, Germany; 9 Institute of Pathology, University of Düsseldorf, Düsseldorf, Germany; 10 Department of Pathology, Technische Universität Dresden, Dresden, Germany; 11 Institute of Pathology, RWTH Aachen University Hospital, Aachen, Germany; 12 Institute of Diagnostic Virology, Friedrich-Loeffler-Institut, Federal Research Institute for Animal Health, Greifswald-Insel Riems, Germany

**Keywords:** Viral infection, Respiratory tract diseases

## Abstract

The rate of SARS-CoV-2 infections in vaccinees has become a relevant serious issue. This study aimed to determine the causes of death, histological organ alteration, and viral spread in relation to demographic, clinical-pathological, viral variants, and vaccine types for deceased individuals with proven SARS-CoV-2 infection after vaccination who died between January and November 2021. Twenty-nine consecutively collected cases were analyzed and compared to 141 nonvaccinated control cases. Autopsies were performed on 16 partially and 13 fully vaccinated individuals. Most patients were elderly and suffered from several relevant comorbidities. Real-time RT-PCR (RT-qPCR) identified a significantly increased rate of generalized viral dissemination within organ systems in vaccinated cases versus nonvaccinated cases (45% vs. 16%, respectively; P = 0.008) mainly with Ct-values of higher than 25 in non-respiratory samples. However, vaccinated cases also showed high viral loads, reaching Ct-values below 10, especially in the upper airways and lungs. This was accompanied by high rates of pulmonal bacterial or mycotic superinfections and the occurrence of immunocompromising factors, such as malignancies, immunosuppressive drug intake, or decreased immunoglobulin levels. All these findings were particularly accentuated in partially vaccinated patients compared to fully vaccinated individuals. The virus dissemination observed in our case study may indicate that patients with an impaired immune system have a decreased ability to eliminate the virus. However, the potential role of antibody-dependent enhancement must also be ruled out in future studies. Fatal cases of COVID-19 in vaccinees were rare and often associated with severe comorbidities or other immunosuppressive conditions.

## Introduction

Vaccination against SARS coronavirus-2 (SARS-CoV-2) combined with contact restrictions is the only way to reduce individual deaths and thus control the ongoing COVID-19 pandemic^1^. Vaccines against SARS-CoV-2 targeting the viral spike protein have been available since the end of 2020. In Europe, four vaccines (BNT162b2, mRNA-1273, AZD1222, and Ad26.COV2.S), which have demonstrated efficacies of up to 95% against COVID-19, have been approved by the European Medicines Agency (EMA) and put into use^2–5^. Next to protection from infections, avoiding severe clinical courses is the main goal of vaccination against SARS-CoV-2. It is anticipated that infection and disease due to SARS-CoV-2 infection may occur despite vaccination, even after the vaccination scheme is completed^6^. However, a distinction must be drawn between “vaccination failure” and “breakthrough infections.” Vaccination failure is usually defined as the failure of the immune system to build effective protection through antibody- and T-cell-based responses against a virus. In contrast, breakthrough infections occur even though the antibody titers achieve sufficient values^7,8^. On 28 October 2021, 1078 such infections with fatal outcomes were recorded in Germany^9^. Another aspect is the protective potential of a partial vaccination after the application of the first dose and the role of different variants of SARS-CoV-2. In a large trial, the efficacy of a single dose of Ad26.COV2.S against moderate and severe COVID-19 was 52% and 64%, respectively, indicating fast immunization in a broad portion of the population^4^. Other studies have reported comparable results^10–12^. A certain level of immune escape of so-called variants of concern (VOCs), for example, the beta variant, has been described by several authors for different vaccines^13–16^.

During the COVID-19 pandemic, autopsies have become remarkably important for understanding the pathophysiology of this new disease. In particular, the viral effects on different organs in severe and lethal cases can, in most cases, only be investigated by thorough autopsies using sophisticated, state-of-the-art diagnostic methods^17,18^. Most relevant COVID-19-associated organ alterations, such as diffuse alveolar damage, endothelitis, and thromboembolic events, have been described based on autopsy results^19–30^. However, despite this high autopsy numbers, especially in Europe and the U.S., reports on autopsies of SARS-CoV-2 breakthrough infections are widely lacking. Currently, only a single case report from Germany of a partially vaccinated case is available ^31^.

This multicenter retrospective study aimed to provide data from a series of fatal cases of COVID-19 after partial and full vaccination. Special attention was paid to the identification of risk factors, the direct causes of death, and viral dissemination.

## Materials and methods

### Case collections

**Fig. 1:**
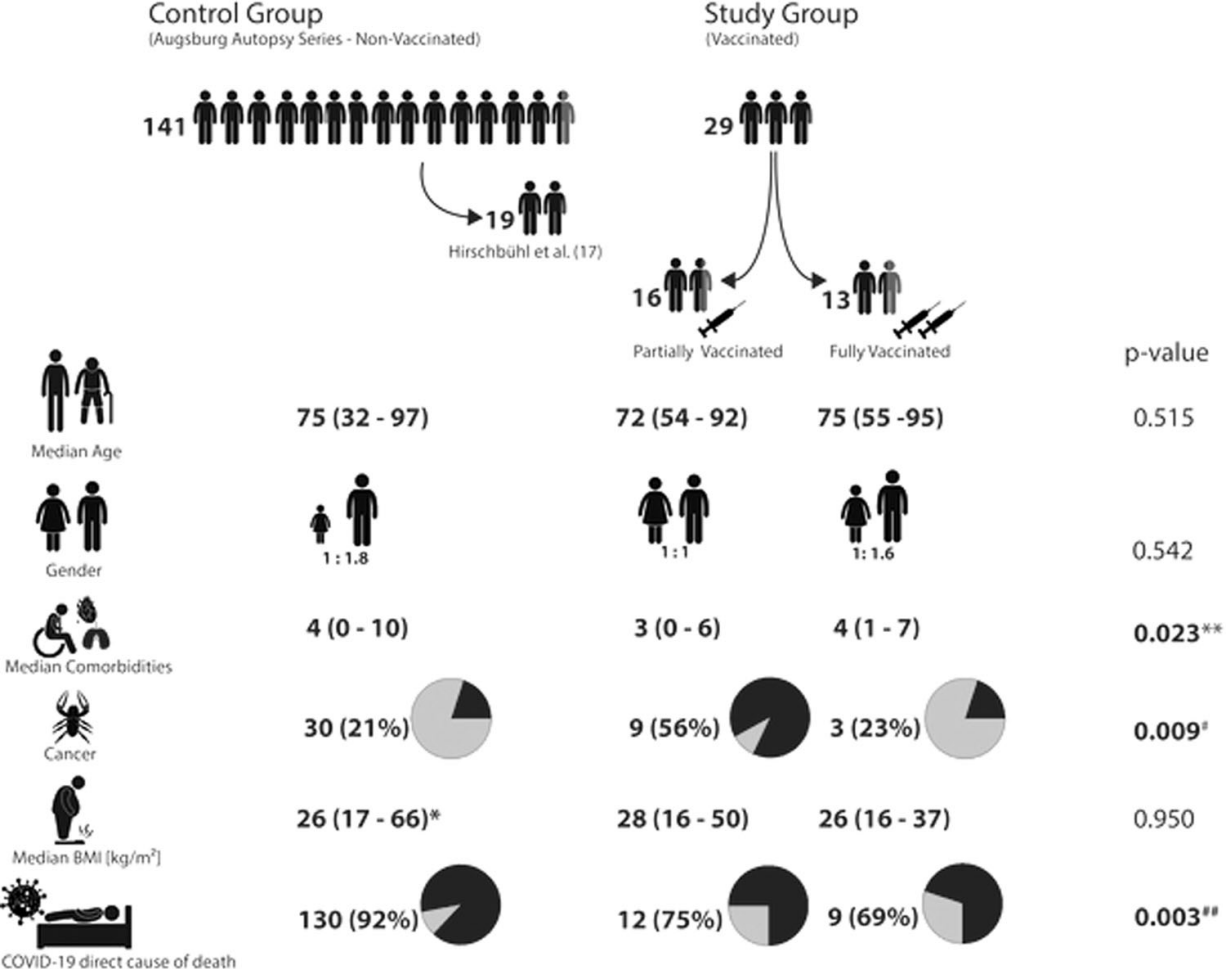
Different study and control groups of the study and basic clinical data. BMI body mass index; * basis are 102 positive cases out of total 129 cases with available information. ** all groups differ significantly from each other (*p* values between 0.014 and 0.047). ^#^ significance occurred between unvaccinated and partially vaccinated cases. ^##^significance occurred between unvaccinated and fully vaccinated cases.

**Fig. 2:**
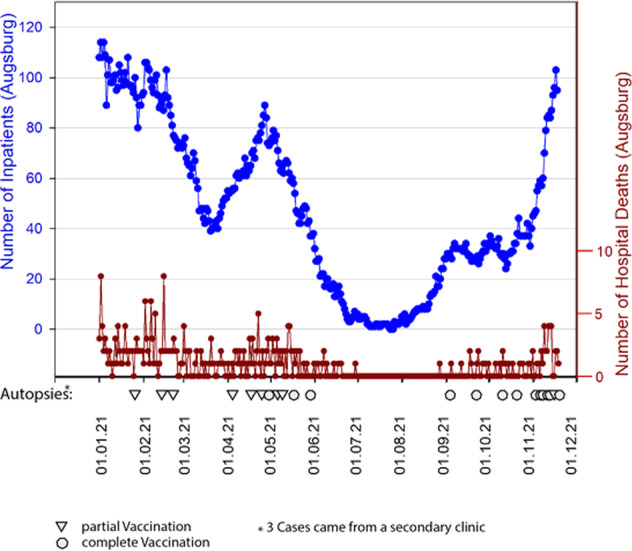
COVID-19 inpatients from 01-2021 to 11-2021 (blue line) and number of COVID-19 deceased during this period (brown line). Triangles and circles indicate autopsies of vaccinated deceased.

The cases for the study group were collected between the end of January and October 2021. Twenty-three of the 29 autopsies were carried out at the University Medical Center of Augsburg. The six remaining cases were included from the University Medical Centers of Düsseldorf (3), Dresden (2), and Tübingen (1). “Full vaccination” was defined as receiving two doses of the vaccine, with the second dose at least 14 days before the onset of symptoms. All autopsies in these cases were performed at the University Hospital Augsburg. Cases that did not fulfill these criteria were classified as “partially vaccinated.” All cases outside Augsburg belong to this group. According to known risk factors for a severe course of COVID-19 (e.g., cardiovascular diseases, diabetes, lung diseases, obesity, cancer, older age, immunosuppression), all but one of the 16 partially vaccinated and all 13 fully vaccinated cases had at least one of the relevant comorbidities. The demographic data, together with clinical-pathological data, are provided in Fig. 1 and Table 1 and Supplementary Tables 1 and 2. The type of infection, breakthrough versus vaccination failure, was determined according to the definition described by Schieffelin et al.^7^. A breakthrough infection was defined as a symptomatic lower respiratory tract infection in a case with at least a low response to full vaccination. Nonvaccinated cases from the Augsburg autopsy series served as controls (*n* = 141). Nineteen of these cases were published previously, providing data on the individual viral spread in fatal cases of COVID-19^17^. The time course of patient numbers and autopsies is given in Fig. 2. Written consent was obtained from the next of kin. This study was approved by the internal review board of the Medical Center Augsburg (BKF No. 2020–18) and the ethics committee of the University of Munich (Project number 20–426, COVID-19 registry of the University Hospital Augsburg, the ethics committee of University Dresden (BO-EK-175052020), the ethics committee of University Düsseldorf (2020-971), and the ethics committee of University Tübingen (236/2021BO2).

**Table 1:** Clinicopathological data.

	Partially vaccinated (*n* = 16)	Fully vaccinated (*n* = 13)	*p* value
Age	72 (32–97)	75 (55–95)	0.272
Gender (f: m)	1: 1	1: 1.6	0.806
Autopsy (partial vs complete)	1: 2.75	1: 1.6	0.689
COVID-19 Cause of death according WHO^a^ (*n*)	15 (94%)	12 (92%)	1.000
Cause of death according autopsy results (condition directly leading to death)	12 (75%)	9 (69%)	1.000
Type of infection^b^ (failure vs. breakthrough)	n.a.	1: 4	
Vaccine (*n*; BT vs. AZ vs. SV)	11: 4: 0^c^	11: 1: 1	0.263
PCR nasopharyngeal swab at diagnosis [Ct value]	n.a.^d^	22 (13–41)	
PCR nasopharyngeal swab at autopsy [Ct value]	18 (9–30)	16 (10–40)	0.974
PCR tissue lowest Ct value [Ct value]^e^	21 (14–31)	21 (17–27)	0.624
Viral dissemination (*n*)	11 (69%)	5 (38%)	0.209
Time from last vaccination to positive test SARS-CoV-2	10 (1–180)	140 (28–283)	**<0.001**
Time from first symptom to death	10 (1–27)	11 (2–24)	0.843
Time from first positive PCR to death	9 (5–25)	9 (1–20)	0.301
Positive SARS-CoV-2 serology - spike (*n*)^f^	4 (80%)	9 (82%)	1.000
SARS-CoV-2 serology - nucleocapsid (*n*)^f^	3 (100%)	5 (45%)	0.209
SARS-CoV-2 lineage- VOC (*n*)	10 (63%)	13 (100%)	**0.020**
IgA-levels [normal: 70–400 mg/dl]^f^	171 (136–255)	98 (58–171)	0.167
IgG-levels [normal: 700–1600 mg/dl]^f^	658 (309–832)	511 (364–820)	0.666
Highest CRP [normal: <0.5 mg/dl]	13 (1–34)	22 (2–35)	0.304
Highest procalcitonin [normal: <0.5 ng/ml]	1 (0–100)	2 (0–100)	0.414
Highest Il-6 [normal: <15 pg/ml]	99 (90–50.000)	506 (16–50.000)	0.281
Malignancies (*n*)	9 (56%)	3 (23%)	0.130
Other comorbidities (*n*)	3 (0–6)	4 (1–7)	**0.047**
BMI [kg/m²]	28 (16–50)	26 (16–37)	0.617
Invasive ventilation (*n*)	5 (31%)	5 (38%)	0.714
Dexamethasone Therapy (*n*)^f^	8 (73%)^f^	7 (54%)	0.423
Acute DAD (*n*)	12 (75%)	9 (69%)	1.000

Aggregated data of main characteristics of partially and fully vaccinated cases (for data of each case see Supplementary Tables 1 and 2). Continuous data are given as median with range in brackets.

Bold values indicate statistical significance *p* < 0.05.

^a^See [Ref.^33^].

^b^See [Ref.^7^].

^c^Vaccine was unknown in one case.

^d^Ct-values have not been determined by the central laboratory during the first half of the year 2021.

^e^The lowest Ct-values were found in lung samples in all cases.

^f^Results are available only in a part of the cases (see Supplementary Tables 1 and 2).

### Autopsy, sample collection, and histology

The autopsy techniques and histology workup have been described previously^17^. Depending on the consent, complete autopsies involving the opening of all body cavities or partial autopsies of differing degrees were performed. In case of partial autopsy, tissue samples of the thoracic and abdominal organs were obtained from epigastric access or using a recently established scopic technique^32^. Regardless of the autopsy technique, tissue samples from organs and soft parts were collected and fixed in 10% buffered formalin for at least 24 h and then embedded in paraffin. Liquid samples, if available, were collected from cerebrospinal fluids and effusions. In cases at the Augsburg Center, nasopharyngeal swabs were also performed. This was done immediately before performing the autopsy.

The causes of death were determined according to the official definition of the WHO^33^, with an indication of the disease underlying the death. Additionally, the immediate cause of death was determined.

### Molecular methods

#### Real-time RT-PCR (RT-qPCR)

The RT-qPCR method that was used has been described recently^17^. RNA was extracted from FFPE sections using the Maxwell® CSC RNA FFPE Kit (AS1360, Promega) and swabs using the Maxwell® 16 LEV Blood DNA Kit (AS1290, Promega) on a Maxwell automatic purification system (Promega Corporation, Madison, WI, USA). In order to verify the efficacy of the sample preparation, an MS2 Phage control was added to the samples before extraction of the RNA. With each extraction run a negative control containing only MS2 Phage was prepared and used as the negative control for RT-qPCR after extraction. RT-qPCR was performed on a QuantStudio 5 Dx real-time PCR instrument (Thermo Fisher, Carlsbad, CA, USA) using the Taq-Path COVID-19 CE-IVD RT-PCR Kit (Thermo Fisher, Pleasanton, TX, USA). The cycle threshold (Ct) values were classified in six categories (<10; 11–17; 18–24; 25–29; 30–40; negative). In cases where whole-genome sequencing of the virus was not available, the variants were determined by PCR-based mutation analysis. Viral dissemination was defined in complete autopsies as RT-qPCR viral RNA detection in at least six of seven locations (lung, heart, central vessel, kidney, liver, spleen, and mediastinal fat). Due to the limited availability of samples from different organs, the definition had to be adapted in partial autopsies. In these situations, the PCR positivity of all samples was assumed to be dissemination. Of note: dissemination in this sense is only a function of the distribution within the organ system but not of the extent of the viral load of the locations.

#### RNA in situ hybridization

RNAscope in situ hybridization (ISH) assays were conducted at the Department of Pathology of the University Hospital of Augsburg to detect SARS-CoV-2 genomic RNA in FFPE tissues. The analysis was performed on respiratory samples with RT-qPCR Ct-values of <25 and on 23 non-respiratory samples of 13 consecutive cases from the beginning of the study (Supplementary Table 3). ISH was performed using SARS-CoV-2 RNA-specific antisense probes designed and synthesized by Advanced Cell Diagnostics (ACD, Palo Alto, CA, USA; Cat. No: 848568). Probes specific to the dihydrodipicolinate reductase B mRNA of Bacillus subtilis (DapB) and peptidylprolyl isomerase B (Hs-PPIB) (ACD, Cat. No: 313908) or ubiquitin C (Hs-UBC) (ACD, Cat. No: 312028) were used as negative and positive controls, respectively, to assess assay specificity and RNA integrity. The RNAscope ISH assays were conducted using the RNAscope 2.5 LS Reagent kit-BROWN (ACD, Cat. No: 322100) on the Leica BOND-RX System (Leica, Germany), following the automated RNAscope protocol optimized for use on this platform. FFPE sections were baked and deparaffinized in the instrument, followed by target retrieval for 25 min at 95 °C in 1X target retrieval solution and protease treatment for 35 min at 40 °C. Subsequently, slides were incubated with the ready-to-use (RTU) target probe mixtures for 2 h at 42 °C, followed by signal amplification with a set of specific amplifiers (AMP1-6). Chromogen detection and hematoxylin counterstaining were performed using a bond polymer refine detection kit (Leica, Cat. No.: DS9800) on the Leica BOND (Leica, Wetzlar, Germany).

#### SARS-CoV-2 sequencing and sequence analysis

The SARS-CoV-2 viral target genome amplicon libraries were constructed using the QIAseq SARS-CoV-2 Primer Panel V1 (Qiagen, Germany), coupled with the QIAseq FX DNA library kit (Qiagen, Germany), following the manufacturer’s protocols. Briefly, 5 µl of total RNA of swab samples of different viral inputs (Ct-value between 18 and 28) was reverse transcribed to synthesize cDNA using random hexamers. Then, 5 µl of cDNA was evenly split into two PCR pools (2.5 µl for each pool) and amplified into 400 bp amplicons using two sets of primers that cover 99% of the entire SARS-CoV-2 genome. The primer panel was designed based on ARTIC V3 primers. PCR was performed according to the manufacturer’s instructions with 35-cycle amplification. After amplification, the contents of the two PCR pools were combined into one single tube for each sample, followed by an AMPure bead cleanup, following the manufacturer’s instructions. The purified amplicons were quantified using the Quantus System (Promega) and normalized for DNA library construction. Enzymatic fragmentation and end repair were performed to generate 250 bp DNA fragments. The fragmentation time was set to 20 min. The AMPure bead cleaned-up DNA libraries were further amplified, i.e., eight cycles for the 40 ng input of amplicons or 20 cycles for the 1.8 ng input of amplicons. The final libraries were quantified by Quantus (Promega) prior to sequencing. Next, the libraries were multiplexed with different barcodes and pooled at 2 nM in equimolar amounts. The pooled libraries were clustered and sequenced on an Illumina MiSeq V2 flow cell at a final concentration of 9 pM (Illumina, Inc., San Diego, CA, USA).

The SARS-CoV-2 whole-genome sequence of some cases was generated using the application of a generic metagenomics workflow^34^ in combination with a capture enrichment procedure using myBaits^35^ or the Ion AmpliSeq SARS‑CoV‑2 Research Panel (ThermoFisher) with 10 µg RNA as input. For the latter application, an Ion Chef instrument was used. After a quality check and quantification, the libraries were pooled and sequenced on an Ion Torrent S5XL instrument (ThermoFisher).

For analysis after the sequencing of each library, FASTQ files were imported into CLC Genomics Workbench version 21.0.1 (Qiagen A/S, Vedbæk, Denmark) with the CLC SARS-CoV-2 workflow. Briefly, reads were imported, trimmed, and mapped to the SARS-CoV-2 reference sequence Wuhan-Hu-1 (MN908947.3). Alternatively, raw data sets were analyzed using the Genome Sequencer Software Suite (version 2.6; Roche, Mannheim, Germany https://roche.com), with default software settings for quality filtering and mapping, and using the reference mentioned above. The SARS-CoV-2 genome sequences generated in this study are available under ENA study accession number PRJEB49094.

#### Phylogenetic analysis of SARS-CoV-2 sequences

Sequences were attributed to SARS-CoV-2 lineages using pangolin (https://pangolin.cog-uk.io/^36^). In addition, the obtained SARS-CoV-2 genome sequences were aligned together and with sequences retrieved from GenBank using MAFFT version 7.388^37^ as implemented in Geneious version 10.2.3 (Biomatters, Auckland, New Zealand). Phylogenetic trees were constructed with PhyML version 3.0^38^, using the GTR + GAMMA + I model with 100 bootstrap replications, and MrBayes version 3.2.6^39^, using the GTR model with eight rate categories and a proportion of invariable sites in the Geneious software package. The Bayesian analysis was performed for 1,000,000 generations and sampled every 1000 generations for four simultaneous chains.

#### Statistics

Depending on group size, categoric data were compared either with Chi-Square or Fisher’s Exact tests. For the comparison of continuous data, the two-tailed Student’s *t* test was used for normally distributed data. Ranked data were compared using the Mann–Whitney Rank Sum test or an analysis of variance (ANOVA) on ranks test. A *p* value of less than 0.05 was considered significant. The association between ranked variables was analyzed using Spearman’s correlation. All tests were performed using the Sigma Plot software package 13.0 (Systat, San Jose, CA, USA).

## Results

### Representativity of the cohort

Given the 303 cases of deceased individuals with COVID-19 during the time of vaccine availability in the Augsburg Center, the 42 (14%) deceased individuals with COVID-19 after vaccination, 23 of which (autopsy rate 55%) are included in this study, indicates that the study cohort is representative.

The University Medical Center Augsburg is the only tertiary medical center in a region with about two million inhabitants. For the city of Augsburg, the rate of completely vaccinated persons is 65% (194,000 persons; status: 4 November 2021)^40^. The 16 deceased cases after full vaccination represent a rate of 0.008%, which is considerably low and in line with a large population-based study carried out in Scotland showing a rate of 0.007% ^41^.

### Partially vaccinated cases

Partial vaccination was present in 16 cases. The median time between vaccination and the first positive PCR test was 10 days (range: 1–24). Eleven patients received the BNT162b2 vaccine (BioNTech), and four were vaccinated with AZD1222 (AstraZeneca). In one case, information regarding the vaccine was not available. There was a nonsignificant trend toward the predominance of females among AZD1222 vaccinated patients (*P* = 0.282). Otherwise, the female and male genders were balanced in this group. No correlation was found between the vaccine and other clinical-pathological data. The viral variants included five cases of non-VOCs (B.1.221, B.1.258, B.1.9.4) and 11 VOCs (ten alpha, one delta; see Table 1, Supplementary Table 1 and Fig. 4 for lineage assignment). This finding reflected the prevalent variants at the respective times of the pandemic (Figs. 2 and 4).

**Fig. 3:**
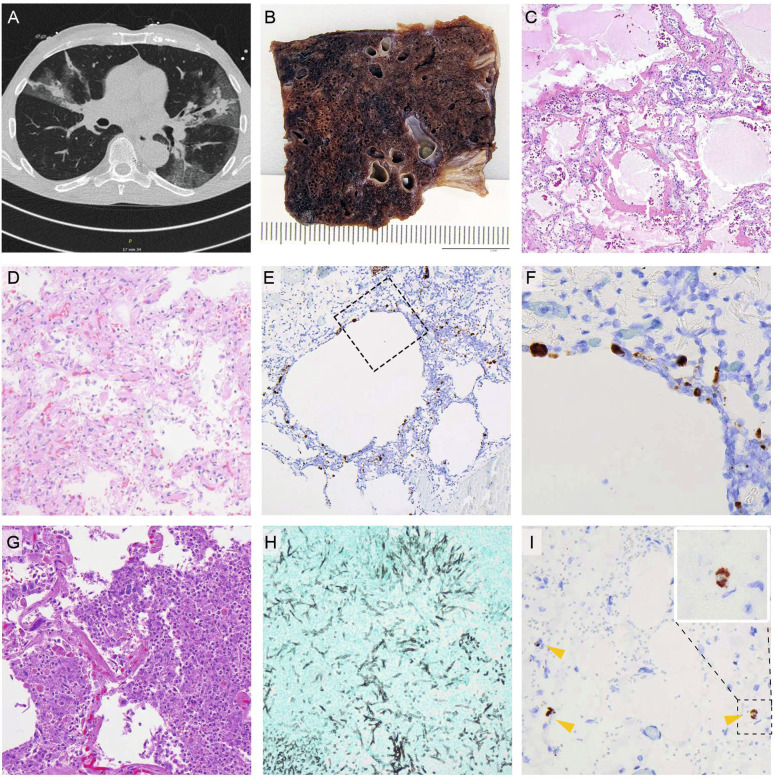
Representative gross and histology images. **A** CT-scan of a COVID-19 pneumonia after single vaccination. **B** Macroscopic image; formalin-fixed; lung parenchyma is widely destroyed with dark areas of hemorrhage and loss of spongious morphology. **C** H&E 40x magnification; acute DAD with prominent hyaline membranes. **D** H&E 200x magnification; organizing DAD with fibroblastic proliferation and loss of alveolar spaces. **E** RNA-ISH 100x magnification; high viral infection of pneumocytes and probably macrophages around emphysematic alveolar structures (**F**) higher magnification of the area in E marked by a square. **G** H&E 400x magnification; acute bacterial pneumonia with dense aggregates of granulocytes within the alveolar spaces. **H** Grocott 200x magnification; Invasive aspergillosis of the lung. **I)** RNA-ISH 300x magnification; infection of three histiocytic cells (arrow heads) within the adventitia of the aorta, insert: higher magnification of the positive cell within the dashed square.

**Fig. 4:**
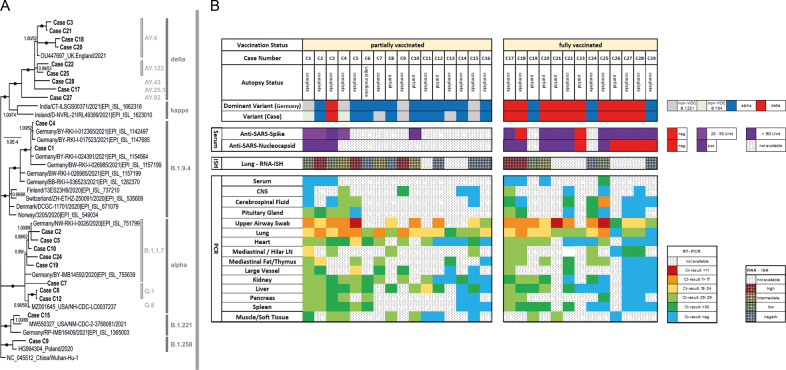
SARS-CoV-2 lineages and viral load data on a case basis. **A** Phylogenetic tree of SARS-CoV-2 lineages including presented cases. Dots indicate bootstrap values of 1.00/100 (MrBayes/Maximum Likelihood). Support values above 50% are given. **B** Autopsy-Status, viral variant lineages and dominant variety in Germany at the time of the individual case, anti-SARS-antibody titer, and viral infection in different organs by RT-qPCR and RNA-ISH (for lungs only). Note: Cases are not sorted in consecutive manner but grade of viral dissemination.

In one case (C6), the SARS-CoV-2 infection was most likely not the cause of death, according to the definition of the WHO. This patient died due to traumatic cerebral bleeding. In the remaining 15 cases, the underlying cause of death based on the WHO definition^33^ was COVID-19. The direct cause of death in three of the 15 cases was cerebral ischemia, cardiac failure, and bleeding, while 12 patients died directly due to severe COVID-19 pneumonia with diffuse alveolar damage (DAD) (Table 1 and Supplementary Table 1). The histological presentation of the cases especially also of the lungs was similar to that of nonvaccinated cases (Fig. 3). RT-qPCR-based detection of SARS-CoV-2 RNA from upper airway swabs revealed low Ct-values (median: 18; range: 9–30), indicating high viral loads. Intraindividual viral dissemination was identified in 11 out of 16 cases (Fig. 4B). This rate was higher than in fully vaccinated cases, with a rate of 38% (*P* = 0.144), but it failed to achieve significance. However, it was significantly higher compared to the 19 previously published nonvaccinated cases of the first wave^17^, with 16% (*P* = 0.002) showing such a dissemination pattern.

In partially vaccinated patients, the lungs were the most affected organs. High viral loads could be detected by RT-qPCR, with a median Ct-value of 21 (range: 14–31), confirmed by RNA-ISH (Fig. 3), which showed a strong correlation with the Ct-values (*R* = 0.819, *P* < 0.0001) in a semiquantitative analysis (Fig. 4). Another remarkable observation in this collection was the high rate of malignancies in their medical history of 56%. Again, this is considerably higher compared to the completely vaccinated cases (3 vs. 10, 23%; *P* = 0.130) and the naïve control cohort (30 vs. 112, 21%; *P* = 0.005).

### Fully vaccinated cases

This study comprised 13 fully vaccinated cases. The median time from the last vaccination to a positive test of SARS-CoV-2 was 140 days (range: 28–283). The vaccines applied in the study groups were BNT162b2 (BioNTech) in 11 cases and AZD1222 (AstraZeneca) and CoronaVac (Sinovac) in one case each. The gender ratio was balanced. No correlation between vaccine type and other clinical findings was observed. SARS-CoV-2 VOCs alpha and delta were the only viral variants present (Table 1 and Supplementary Table 2 and Figs. 2 and 4).

According to the WHO classification^33^, all but one patient died due to COVID-19. The non-COVID-19 patient suffered from myocardial infarction and renal abscesses. The SARS-CoV-2 RT-qPCR during routine testing on admission was positive, with a very high Ct-value of 41. The condition leading directly to death, (not following the definition of the WHO), was other than COVID-19 (Table 1 and Supplementary Table 2, Fig. 1) at a rate of 31% (4 of 13). The corresponding rates were 25% (4 of 16; *P* = 1.000) and 8% (11 of 141; *P* = 0.029) in partially vaccinated cases and nonvaccinated cases, respectively.

The type of infection (“real breakthrough” versus “vaccination failure,” according to Schieffelin et al.^7^) could be classified in 11 of the 13 cases in which serology for SARS-CoV-2 spike-specific antibodies was positive. Five of these cases revealed high anti-spike titers (>2500 U/ml), while others showed only moderate levels (154–407 U/ml). In the remaining two cases, no antibodies against the spike protein were detected (Table 1 and Supplementary Table 2). According to these results, eight cases were classified as breakthrough infections, whereas two cases represented vaccine failures. In one case, only an asymptomatic infection was obtained. The SARS-CoV-2 nucleocapsid antibody serology revealed negative results (COI < 1) in six of 11 cases, which correlated significantly with the occurrence of viral dissemination (*P* = 0.015).

In contrast to the partially vaccinated cases, the viral spread in fully vaccinated cases was restricted to the upper airways and lungs in eight of the 13 cases, whereas viral dissemination throughout the organ system was seen in five cases. Again, histological changes in the organs were similar to nonvaccinated cases, with relevant impairment of the lungs. The median RT-qPCR Ct-value of the lungs was 23 (range: 17–27), similar to the partially vaccinated cases (median 21, range 14–31).

### RNA-ISH from non-respiratory samples

RNA-ISH from 23 non respiratory samples (Supplementary Table 3) revealed positive results in only five samples. Positive signals were restricted to 1–4 single cells which are very likely histiocytic cells (Fig. 3I). Epithelial cells or soft tissue cells were not infected. There was a significant correlation between the Ct-values and the corresponding RNA-signal (Correlation: −0.453; *P* = 0.03).

### RT-PCR from blood serum samples

To address the question of a potential role of viremia, seven blood serum samples which were collected 48 h maximum before death were analyzed for SARS-CoV-2-RNA by RT-PCR (Fig. 4B). Positivity with a Ct-value of 30 was found only in one case (C25) that was classified as non-disseminated. Here, an exceptionally low Ct-value in the nasopharyngeal smear was noticeable and a short time between the first positive PCR-test and the death of 6 days indicating an early phase of the disease with high viral replication.

### Sequence analysis

Whole-genome sequencing was conducted for all cases with sample material having proper Ct-values, except C6, C11, C13, C14, C16, C23, C26, C29. In most cases, one sample was subjected to sequencing. For some cases, we could investigate several samples: C1 (*n* = 4), C17 (*n* = 2), C18 (*n* = 2), C19 (*n* = 2), C25 (*n* = 2). Sequences of corresponding samples were identical to each other.

## Discussion

To the best of our knowledge, this is the first series of autopsies of fatal cases of COVID-19 in SARS-CoV-2-vaccinated individuals. The lack of reliable studies and data make it difficult to assess the situation of vaccinated individuals. Therefore, we started to assess viral dissemination in the context of demographic and clinical data to identify potential factors that foster a fatal course of COVID-19 in vaccinees. The aim of this study was to investigate a cohort of 29 fatal COVID-19 cases in vaccinees by collecting all available metadata including SARS-CoV-2 antibody testing and by using necropsy, in situ hybridization, RT-qPCR analysis, and whole-genome sequencing to analyze the course of infection, allowing a substantiated disease and strain characterization.

The focus was on the comparison between partially vaccinated (vaccination interval not completed) and fully vaccinated cases (vaccination interval completed). Moreover, a collection of 141 consecutive cases from nonvaccinated individuals from the Augsburg autopsy series served as controls. Overall, the cases in vaccinees represent about one-third of all deceased in the Augsburg medical center, showing a similar but not identical demographic feature, with a slightly lower proportion of women and a slightly higher age compared to the total collective. All fully vaccinated cases came from the University Medical Center Augsburg, while six of the 16 partially vaccinated cases were contributed by other academic centers.

This study includes two fundamentally different post-vaccination situations, i.e., with partial and full vaccinations. In fully vaccinated cases, the type of infection was classified according to Schieffelin et al.^7^, taking so-called “vaccine nonresponders” into account. However, in our study group of fully vaccinated cases, real “breakthrough infections” occurred in the majority of individuals, and only two of ten cases were defined as likely “vaccination failure,” which therefore might play a limited role in lethal infections. For vaccination failure, it has to be clarified whether it was a primary failure (e.g., nonresponders, application errors, etc.) or loss of vaccination response over time, as recently described in Israel^42^. In our study group, based on serological data, a primary failure due to nonresponse, e.g., during steroid treatment, is the most likely cause in both described cases (C17) (C29)^43^.

The macroscopic and histomorphological findings in the partially vaccinated deceased individuals were similar to the findings in the nonvaccinated cases. Most patients died due to COVID-19 pneumonia with typical DAD. Superinfections (Supplementary Tables 1 and 2) occurred at a relatively high frequency (11 of 29), including aspergillosis (four cases). This is considerably more frequent compared to our previous results^17^ but rarer than reported in deceased patients after long-term treatment^44^. Other organs showed no histological alterations that could be associated with SARS-CoV-2 infection. However, a high rate of viral dissemination detected by RT-qPCR within the organ system was an unanticipated result in this study, which was especially accentuated in the partially vaccinated compared to fully vaccinated cases (11 of 16 vs. five of 13, respectively; *P* = 0.144). In several cases, RT-qPCR identified the RNA of SARS-CoV-2 in all investigated samples, including cerebrospinal fluid, CNS, and soft tissues. This is in strong contrast to a previously published collection of the Augsburg series of nonvaccinated lethal SARS-CoV-2 infections, in which the frequency of viral dissemination was rare, with a rate of only 16% (three of 19)^17^ instead of 69%. In this context, it seems especially important to compare the results of different cohorts within the same analytic system. Other authors have reported results we classify in this study as “disseminated” at high frequencies^45,46^, but they used different settings and methods.

The low Ct-values of nasopharyngeal swabs and lung samples, the latter with abundant viral detection by RNA-ISH, underline high viral loads in vaccinated deceased individuals, again with accentuation in partially vaccinated individuals. However, the previous series^17^ did not include VOCs. Therefore, it cannot be ruled out that the reported increased viral loads are, in part, also a consequence of the respective circulating viral variants. However, because we also found this effect in non-VOC vaccinees and observed anecdotical restricted dissemination of VOCs, including the delta and gamma variants in non-vaccinees (data not shown), it is probable that the dissemination phenotype observed here is not related to the given variant. A recently published study showed that a single shot of AZD1222 or BNT162b2 exhibited a relevant protective effect against infection with SARS-CoV-2^47^. However, this does not equate to complete protection, and individual fatal courses (e.g., also related to preexisting disease conditions) are supported by our data. The PCR-based viral detection in samples from other locations than lung could be confirmed only in part by RNA-ISH in a few cases. This is most likely due to its reduced sensitivity compared to the PCR. We and others have shown this previously^17,18,48^. The fact that we could demonstrate a correlation between the Ct-values from the lung and the non-respiratory samples with the corresponding RNA-ISH results support this interpretation. In these cases, viral ISH signals were not found in parenchymal or soft tissue cells but in histiocytic cells. This is in concordance with the lack of a relevant parenchymal impairment based on the histomorphological findings. Viremia investigated by RT-PCR analysis of blood serum was detected only in one out of seven cases. This single case showed an exceedingly low Ct-value of the nasopharyngeal swab and was classified as non-disseminated. The six samples with negative serum-PCR-results were obtained from cases classified as disseminated. Serum testing by PCR has recently been shown being efficient to detect SARS-CoC-2 viremia providing a high prognostic relevance^49,50^. Even if we cannot rule out viremia completely it seems unlikely to be the underlying mechanism.

Despite the lack of COVID-19-specific histopathological alterations of non-respiratory organs the distribution of the virus in concert with low Ct-values in the nasopharyngeal swabs and lung samples remains a relevant result in the majority of the cases in this series.

Two major contrary theses could explain this viral spread: 1) the vaccination itself and 2) the constitution of the individual. The first is mediated by antibody-dependent enhancement (ADE)^51–55^, which is known from other viral infections, such as dengue^56^, Ebola^57^, and HIV^58^. In ADE, antibodies do not eliminate the virus or do so only to a reduced extent; instead, they promote viral uptake into the host’s cells. Virus-bound IgG is carried into immune cells by Fc-receptor-mediated internalization. The extent to which ADE plays a role in coronavirus infections is unclear. Reports advocating the existence of ADE in coronavirus infections are based on experiments using cell cultures^59,60^ or animal models^61^. However, there is currently no evidence indicating that ADE is a relevant mechanism counteracting the protective role of anti-spike protein antibodies generated by vaccines in humans. A large study of 20,000 patients receiving COVID-19 convalescent plasma reported no safety concerns^62^, which can also be considered a powerful argument against the relevant role of ADE in humans. Currently, no assays or biomarkers have been established to prove ADE in vivo. Immune cell infiltration, including eosinophils indicating an adverse immune reaction, is restricted to T-helper cell-mediated responses and is not related to ADE ^63^.

Focusing on potential patient-related factors, the immune system is of major interest in the context of failing viral elimination. Both collections in this study are characterized by a high median age and a high rate of potentially immune compromising conditions, such as cancer history (12 individuals), intake of immunosuppressive drugs (three individuals), asplenia (one individual), or decreased immunoglobulin levels (three individuals). One or more of these conditions were found in 69% and 40% of partially and fully vaccinated patients, respectively. A very recent clinical study underlines the role of immune compromission^64^. The finding that negative nucleocapsid antibody testing was associated with strongly increased or generalized viral dissemination in fully vaccinated cases (Table 1 and Supplementary Table 2) further supports the hypothesis that the immune system of these patients was no longer able to elicit a primary response versus the SARS-CoV-2 nucleocapsid protein, while spike-specific antibodies were often present or even boosted to high titers (Table 1 and Supplementary Table 2). In terms of cancer, a recently published study showed that malignancies are important risk factors for COVID-19, hospitalization, and death^65^. One explanation for this finding is the lower rate of seroconversion after vaccination of cancer patients in general as a result of immunosuppression (disease and therapy)^66,67^. The same is true for immunosuppressive antirheumatic drugs ^43^.

A general limitation of autopsy studies such as ours is the rather small case number. In an ongoing pandemic, inhomogeneities regarding the included variants might further weaken the study. Nevertheless, the consecutively collected cases with an appropriate rate can be assumed to be representative enough to draw relevant conclusions.

Overall, this is the first series of fatal courses of COVID-19 after vaccination that was analyzed in detail using a broad range of diagnostic techniques. As a major outcome, it can be concluded that most of the deceased were elderly patients with a high number of comorbidities. Lethal SARS-CoV-2 infection in vaccinated individuals therefore seems to be a very rare event and is mainly connected with high age and additional underlying factors, such as chronic diseases. A high viral infection, both in terms of the spread within the organ system and viral load in the respiratory system (detected by RT-qPCR), together with high rates of immunocompromising conditions, are the most striking findings of this study, which were accentuated in cases with an incomplete vaccination status.

## Supplementary information

Supplementary Tables 1 and 2

## Data Availability

The very most data are already provided within this article. Further data can be requested from the corresponding author as far as the privacy of the deceased and relatives is not violated.
