# Supplementary material for: High viral loads: what drives fatal cases of COVID-19 in vaccinees? – an autopsy study
Source: Mod Pathol. 2022 Apr 1;35(8):1013–21. doi: 10.1038/s41379-022-01069-9 (PMC8974809; doi:10.1038/s41379-022-01069-9)
Supplement: Supplementary file 1 — Supplementary Tables 1 and 2 [file 41379_2022_1069_MOESM1_ESM.docx]

**High viral loads: what drives fatal cases of COVID-19 in vaccinees?**

**– an autopsy study**

**Running Title: Autopsies in COVID-19 vaccinees**

Klaus Hirschbühl*, MD; Tina Schaller*, MD; Bruno Märkl, MD; Rainer Claus, MD; Eva Sipos, PhD; Lukas Rentschler, MD, Andrea Maccagno, MD, Bianca Grosser, MD, Elisabeth Kling, MD; Michael Neidig, MD, Thomas Kröncke, MD; Oliver Spring, MD; Georg Braun, MD; Hans Bösmüller, MD; Maximilian Seidl, MD; Irene Esposito, MD; Jessica Pablik, MD; Julia Hilsenbeck, PhD; Peter Boor, MD, PhD; Martin Beer, DVM; Sebastian Dintner*, PhD; Claudia Wylezich, PhD*

^*^ these authors contributed equally

**Supplementary Information**

**Supplementary Table 1**

| **Case Number** | **C1** | **C2** | **C3** | **C4** | **C5** | **C6** | **C7** | **C8** | **C9** | **C10** | **C11** | **C12** | **C13** | **C14** | **C15** | **C16** |
| --- | --- | --- | --- | --- | --- | --- | --- | --- | --- | --- | --- | --- | --- | --- | --- | --- |
| **Age Decade** | 6 | 8 | 8 | 10 | 7 | 7 | 6 | 8 | 7 | 9 | 6 | 8 | 8 | 8 | 10 | 7 |
| **Gender** | male | male | female | female | male | female | male | male | female | male | female | male | female | male | female | female |
| **Autopsy** | complete | complete | complete | complete | complete | Legal medicine | complete | partial | complete | partial | complete | partial | complete | complete | complete | complete |
| **Cause of death according WHO*** | COVID-19 | COVID-19 | COVID-19 | COVID-19 | COVID-19 | Not COVID-19 | COVID-19 | COVID-19 | COVID-19 | COVID-19 | COVID-19 | COVID-19 | COVID-19 | COVID-19 | COVID-19 | COVID-19 |
| **Cause of death according autopsy results (condition directly leading to death)** | COVID-19 pneumonia | COVID-19 pneumonia | COVID-19 pneumonia | COVID-19 pneumonia | COVID-19 pneumonia | traumatic (cerebral bleeding) | COVID-19 pneumonia | COVID-19 pneumonia | COVID-19 pneumonia | COVID-19 pneumonia | COVID-19 pneumonia | COVID-19 pneumonia | cardiac failure | hemorrhagic shock | COVID-19 pneumonia | cerebral ischemia |
| **Vaccination status** | partial | partial | partial | 2-times** | partial | partial | partial | partial | partial* | partial | partial | partial | partial | partial | partial | partial |
| **Vaccine** | BNT162b2 | BNT162b2 | BNT162b2 | BNT162b2 | BNT162b2 | AZD1222 | BNT162b2 | AZD1222 | n.a. | BNT162b2 | AZD1222 | BNT162b2 | BNT162b2 | BNT162b2 | BNT162b2 | AZD1222 |
| **Nasopharyngeal swab at diagnosis [Ct value]** | n.a. | n.a. | n.a. | n.a. | n.a. | 18 | n.a. | n.a. | n.a. | n.a. | n.a. | n.a. | n.a. | n.a. | n.a. | n.a. |
| **Nasopharyngeal swab at autopsy [Ct value]** | 16 | 22 | 17 | 18 | 9 | 18 | positive | 21 | positive | 14 | 25 | 13 | positive | 30 | 22 | 26 |
| **PCR tissue lowest Ct value  [Ct value (Organ)]** | 18 (lung) | 16 (lung) | 22 (lung) | 17 (lung) | 15 (lung) | 27 (lung) | 14 (lung) | 27 (lung) | 14 (lung) | 19 (lung) | 21 (liver) | 20 (lung) | 31 (lung) | 26 (lung) | 26 (lung) | 24 (lung) |
| **Viral dissemination** | dis | dis | dis | dis | dis | dis | dis | dis | dis | dis | dis | non-dis | non-dis | non-dis | non-dis | non-dis |
| **Time from last vaccination to positive test SARS-CoV-2** | 1 | 13 | 180 | 10 | 3 | 10 | 13 | 21 | 4 | n.a. | 11 | 6 | n.a. | 5 | 24 | 10 |
| **Time from first symptom to death** | 5 | 6 | 27 | 7 | 5 | 9 | 12 | 25 | 1 | 6 | 20 | 11 | 3 | 13 | 27 | 25 |
| **Time from first positive PCR to death** | 9 | 5 | 12 | 13 | 5 | 9 | n.a. | 25 | n.a. | 5 | 9 | 11 | n.a. | 9 | 15 | 23 |
| **SARS-CoV-2 serology - spike [normal: <0.8 U/ml]** | 21 | 45 | >2500 | 34 | n.a. | n.a. | n.a. | n.a. | n.a. | < 0.8 | n.a. | n.a. | n.a. | n.a. | n.a. | n.a. |
| **SARS-CoV-2 serology - nucleocapsid [normal: < COI 1]** | 14 | 1.3 | 2.8 | n.a. | n.a. | n.a. | n.a. | n.a. | n.a. | n.a. | n.a. | n.a. | n.a. | n.a. | n.a. | n.a. |
| **VOC** | - | alpha | delta | - | alpha | alpha | alpha | alpha | - | alpha | alpha | alpha | - | alpha | - | alpha |
| **SARS-CoV-2 Lineage** | B.1.9.4 | B.1.1.7 | B.1.617.2 | B.1.9.4 | B.1.1.7 | B 1.1.7 | B.1.1.7 | B.1.1.7 | B.1.258 | B.1.1.7 | B.1.1.7 | B.1.1.7 | n.a. | B.1.1.7 | B.1.221 | B.1.1.7 |
| **IgA-levels [normal: 70 – 400]** | 255 mg/dl | n.a. | 250 mg/dl | 159 mg/dl | n.a. | n.a. | n.a. | n.a. | n.a. | 136 mg/dl | n.a. | 169 mg/dl | n.a. | n.a. | 172 mg/dl | n.a. |
| **IgG-levels [normal: 700 - 1600]** | 556 mg/dl | n.a. | 832 mg/dl | 309 mg/dl | n.a. | n.a. | n.a. | n.a. | n.a. | 756 mg/dl | n.a. | 681mg/dl | n.a. | n.a. | 635 mg/dl | n.a. |
| **Highest CRP (normal: <0.5)** | 42.0 mg/dl | 21.0 mg/dl | 13.4 mg/dl | 7.0 mg/dl | 8.0 mg/dl | 1.3 mg/dl | n.a. | 34.0 mg/dl | n.a. | 13.0 mg/dl | n.a. | 15.0 mg/dl | n.a. | n.a. | 9.0 mg/dl | n.a. |
| **Highest procalcitonin (normal: <0.5)** | >100 ng/ml | n.a. | 0,2 ng/ml | 1.0 ng/ml | 0.2 ng/ml | 0.3 ng/ml | n.a. | 4.0 ng/ml | n.a. | 0.5 ng/ml | n.a. | 0.4 ng/ml | n.a. | n.a. | <0.5 ng/ml | n.a. |
| **Highest Il-6 (normal: <15)** | >50000 pg/ml | n.a. | 94 pg/ml | 95 pg/ml | n.a. | n.a. | n.a. | 1180 pg/ml | n.a. | 99 pg/ml | n.a. | 175 pg/ml | n.a. | n.a. | 90 pg/ml | n.a. |
| **Malignancy** | none | none | breast cancer | chronic lymphocytic leukemia | metastatic prostatic cnacer | none | MGUS | none | none | renal cell cancer | none | nasopharyngeal cancer | none | gastric cancer | lung cancer, history of colon cancer | multiple myeloma |
| **Other comorbidities** | diabetes, hypertension, hyperlipidemia, mental retardation | COPD, diabetes, hypertension, cardiomyopathia | cerebral infarction, aortic valvular stenosis, heart failure, hypertension, COPD, | aortic valve stenosis, pulmonary hypertension, osteoporosis | arteriosclerosis | hyperlipidemia, arthritis (Methotrexate) | lichen myxoedematosus | coronary artery disease, diabetes, hypertension, peripheral artery disease | hypertension, epilepsia, cerebral infarction, dementia | hypertension | history of thrombosis and lung embolism | none | schizophrenia, left Ovariosalpingectomy splenectomia | coronary artery disease, myocardial infarction, atrial fibrilation, chronic kidney injury, hypertension, cerebral infarction | peripheral artery disease, asthma, congestive heart failure, mitral valve stenosis | hypertension |
| **BMI** | 28 | 50 | 39 | 16 | 25 | 28 | 34 | 17 | n.a. | 34 | 26 | 25 | n.a. | 27 | 23 | 28 |
| **Invasive ventilation** | yes | yes | no | no | no | no | yes | no | no | no | no | no | no | yes | no | yes |
| **Dexamethasone** | yes | yes | yes | no | yes | no | n.a. | yes | n.a. | yes | n.a. | yes | n.a. | n.a. | no | yes |
| **Changes in lung parenchyma** | acute and organizing DAD, small areas with acute pneumonia | acute DAD with focal signs of organization | acute DAD, hemorrhage, congestion, acute pneumonia, aspergillosis | acute and organizing DAD | acute DAD | no DAD, emphysema, mild edema | no DAD, severe congestion, edema, fibrosis, emphysema | acute and organizing DAD, aspergillosis | acute DAD, severe congestion, acute pneumonia | acute DAD | acute DAD, acute pneumonia, organizing pneumonia | acute and organizing DAD | no DAD, congestion, emphysema | acute DAD, hemorrhage, congestion, acute pneumonia | acute DAD, severe acute pneumonia | organizing pneumonia, microthrombi |

Data of the partially vaccinated cases; n.a. = not applicable/available; VOC = variant of concern, DAD = diffuse alveolar damage; COPD = chronic obstructive pulmonary disease, MGUS = ... Monoclonal gammopathy of undetermined significance;*according to WHO 2020 ^33^; **according to Schieffelin *et al. ^7^*

**Supplementary Table 2**

| **Case Number** | **C17** | **C18** | **C19** | **C20** | **C21** | **C22** | **C23** | **C24** | **C25** | **C26** | **C27** | **C28** | **C29** |
| --- | --- | --- | --- | --- | --- | --- | --- | --- | --- | --- | --- | --- | --- |
| **Age Decade** | **8** | **9** | **9** | **9** | **8** | **8** | **8** | **7** | **7** | **8** | **8** | **6** | **10** |
| **Gender** | **male** | **male** | **female** | **female** | **female** | **male** | **male** | **female** | **male** | **male** | **male** | **male** | **female** |
| **Autopsy** | **complete** | **complete** | **partial** | **partial** | **partial** | **complete** | **partial** | **complete** | **complete** | **partial** | **complete** | **complete** | **complete** |
| **Cause of death according WHO*** | **COVID-19** | **COVID-19** | **COVID-19** | **COVID-19** | **COVID-19** | **COVID-19** | **COVID-19** | **COVID-19** | **COVID-19** | **COVID-19** | **COVID-19** | **COVID-19** | **not COVID-19** |
| **Cause of death according autopsy results (condition directly leading to death)** | **COVID-19 pneumonia and myocardial infarction** | **COVID-19 pneumonia and cardiac failure** | **respiratory failure**** | **COVID-19 pneumonia** | **Myocardial infarction or pulmonary embolism C19 associated** | **sepsis** | **COVID-19 pneumonia** | **aspiration pneumonia** | **COVID-19 pneumonia** | **COVID-19 pneumonia** | **COVID-19 pneumonia** | **COVID-19 pneumonia** | **myocardial infarction and nephric abscess** |
| **Type of infection***** | **breakthrough** | **vaccination failure** | **n. d.** | **breakthrough** | **breakthrough** | **breakthrough** | **vaccination failure** | **n. d.** | **breakthrough** | **breakthrough** | **breakthrough** | **breakthrough** | **asymptomatic infection** |
| **Vaccination status** | **complete** | **complete** | **complete** | **complete** | **complete** | **complete** | **complete** | **complete** | **complete** | **complete** | **complete** | **complete** | **complete** |
| **Vaccine** | **BNT162b2** | **BNT162b2** | **AZD1222** | **BNT162b2** | **BNT162b3** | **BNT162b2** | **Sinovac** | **BNT162b2** | **BNT162b2** | **BNT162b2** | **BNT162b2** | **BNT162b2** | **BNT162b2** |
| **PCR nasopharyngeal swab at diagnosis [Ct value]** | **n.a.** | **30.3** | **n.a.** | **34.5** | **13** | **n.a.** | **n.a.** | **n.a.** | **n.a.** | **n.a.** | **n.a.** | **22** | **41** |
| **PCR nasopharyngeal swab at autopsy [Ct value]** | **14** | **13** | **14** | **18** | **10** | **n.a.****** | **n.a.** | **11** | **neg******* | **34** | **20** | **18** | **40** |
| **PCR tissue lowest Ct value  [Ct value (organ)]** | **18 (lung)** | **17 (lung)** | **20 (lung)** | **21 (lung)** | **25 (lung)** | **27 (lung)** | **27 (lung)** | **23 (lung)** | **17 (lung)** | **neg** | **20 (lung)** | **27 (lung)** | **neg.** |
| **Viral dissemination** | **dis** | **dis** | **dis** | **dis** | **dis #** | **non-dis** | **non-dis** | **non-dis** | **non-dis ##** | **non-dis** | **non-dis** | **non-dis** | **non-dis ###** |
| **Time from last vaccination to positive test SARS-CoV-2** | **n.a.** | **249** | **58** | **283** | **150** | **225** | **28** | **105** | **100** | **150** | **140** | **140** | **120** |
| **Time from first symptom to death** | **12** | **5** | **11** | **8** | **2** | **10** | **16** | **5** | **16** | **24** | **18** | **21** | **4** |
| **Time from first positive PCR to death** | **4** | **5** | **10** | **9** | **2** | **9** | **13** | **2** | **6** | **20** | **18** | **16** | **1** |
| **SARS-CoV-2 serology - spike [normal: <0.8 U/ml]** | **407** | **<0.8** | **n.a.** | **>2500** | **278** | **> 2500** | **< 0.8** | **n.a.** | **> 2500** | **223** | **>2500** | **154** | **> 2500** |
| **SARS-CoV-2 serology - nucleocapsid [normal: < COI 1]** | **neg.** | **neg.** | **n.a.** | **neg.** | **neg.** | **neg.** | **2.89** | **n.a.** | **neg.** | **33** | **21.6** | **11.1** | **120** |
| **VOC** | **delta** | **delta** | **alpha** | **delta** | **delta** | **delta** | **delta** | **alpha** | **delta** | **delta** | **delta** | **delta** | **alpha** |
| **SARS-CoV-2 lineage** | **AY.25.1** | **AY.4** | **B.1.1.7** | **AY.4** | **AY.4** | **AY.122** | **n.a.** | **B. 1.1.7** | **AY.122** | **n.a.** | **AY.92** | **AY.43** | **B.1.1.7** |
| **IgA-levels [normal: 70 – 400]** | **n.a.** | **n.a.** | **98 mg/dl** | **n.a.** | **n.a.** | **58 mg/dl** | **n.a.** | **n.a.** | **n.a.** | **171 mg/dl** | **n.a.** | **n.a.** | **n.a.** |
| **IgG-levels [normal: 700 - 1600]** | **n.a.** | **n.a.** | **511 mg/dl** | **n.a.** | **n.a.** | **364 mg/dl** | **n.a.** | **n.a.** | **n.a.** | **820 mg/dl** | **n.a.** | **n.a.** | **n.a.** |
| **Highest CRP (normal: <0.5)** | **35.0 mg/dl** | **17.7 mg/dl** | **12.0 mg/dl** | **7.4 mg/dl** | **1.9 mg/dl** | **19.9 mg/dl** | **26.5 mg/dl** | **n.a.** | **34.4 mg/dl** | **34.2 mg/dl** | **26.0 mg/dl** | **23.8 mg/dl** | **20.6 mg/dl** |
| **Highest procalcitonin (normal: <0.5)** | **50.0 ng/ml** | **n.a.** | **0.16 ng/ml** | **0.1 ng/ml** | **n.a.** | **0.9 ng/ml** | **2.1 ng/ml** | **n.a.** | **2.2 ng/ml** | **2.2 ng/ml** | **21.0 ng/ml** | **0.7 ng/ml** | **>100 ng/ml** |
| **Highest Il-6 (normal: <15)** | **914 pg/ml** | **n.a.** | **16 pg/ml** | **n.a.** | **n.a.** | **196 pg/ml** | **>50.000 pg/ml** | **n.a.** | **1720 pg/ml** | **534 pg/dl** | **477 pg/dl** | **283 pg/ml** | **n.a.** |
| **Malignancy** | **none** | **none** | **MPN** | **none** | **none** | **none** | **none** | **none** | **none** | **none** | **prostate cancer** | **none** | **breast cancer** |
| **Other comorbidities** | **diabetes, renal failure, dialysis, hypertension, arterisclerosis, cerebral infarction** | **severe peripheral artery disease , hypertension, diabetes, chronic renal failure, COPD, Prednisolone therapy** | **hypertension, arteriosclerosis, asthma** | **COPD, heart failure, hypertension, renal failure, dementia, diabetes, pulmonary embolism** | **COPD, coronary heart disease, history of temporal arteritis and hashimoto thyreoiditis** | **myocardial Infarction, hypertension, diabetes, cachexia, dementia** | **hypertension, heart failure, coronary heart disaease, myocardial infarction, renal failure, sleep apnea** | **moderate arteriosclerosis** | **coronary heart disaease, myocardial infarction, hypertension, pulmonary hypertension, cachexia** | **hypertension, coronary heart disease, diabetes, renal failure** | **diabetes, hypertension,** | **rheumatoid arthritis (Adalimumab Methotrexate, Prednisolone), history of tuberculosis,**  **COPD, history of influenza** | **chronic renal failure** |
| **BMI** | **37** | **25** | **24** | **35** | **25** | **16** | **35** | **n.a.** | **16** | **31** | **28** | **27** | **25** |
| **Invasive ventilation** | **no** | **no** | **no** | **no** | **no** | **no** | **yes** | **no** | **yes** | **yes** | **yes** | **yes** | **no** |
| **Dexamethasone** | **yes** | **no** | **no** | **yes** | **no** | **no** | **yes** | **no** | **yes** | **yes** | **yes** | **yes** | **no** |
| **Changes in lung parenchyma** | **acute DAD** | **mild acutue DAD, acute pneumonia, aspergillosis, severe emphysema, severe congestion** | **no DAD, congestion of blood vessels** | **mild acute DAD and acute pneumonia** | **mild unspecific alterations, no DAD** | **acute pneumonia, very mild acute DAD, marked mixed pneumoconiosis** | **mild acute DAD, congestion** | **emphysema, acute pneumonia** | **acute/organzing DAD severe emphysema, acute pneumonia** | **moderate acute DAD** | **acute/organizing DAD** | **organizing DAD with residual acute DAD, aspergillosis** | **UIP, no DAD** |

n.d. = not done; n.a. = not applicable/available; dis = dissemination; DAD = diffuse alveolar damage; UIP = usual interstitial pneumonia; VOC = variant of concern; COPD = chronic obstructive pulmonary disease. * according to WHO 2020 ^33^; ** only mild changes in lungs, no proven other cause of death, but only partial autopsy; *** according to Schieffelin *et al.* ^7^; **** tracheal: 17; ***** tracheal: 14; # only three values, but soft tissue positive; ## but cerebrospinal fluid positive; ### only nasopharyngeal swab positive

**Supplementary Table 3: SARS-CoV-2 RNA-ISH of non-respiratory samples**

CT = Ct-Value; RNA = semiquantitative scoring of RNA-ISH: 0 = negative, 1 = week in single cells, 2 moderate in single cells. Note: No signals were identified in epithelial or soft tissue cells but only in cells that were classified as histiocytes.
